# Recurrence-Based Synchronization Analysis of Weakly Coupled Bursting Neurons under External ELF Fields

**DOI:** 10.3390/e24020235

**Published:** 2022-02-03

**Authors:** Aissatou Mboussi Nkomidio, Eulalie Ketchamen Ngamga, Blaise Romeo Nana Nbendjo, Jürgen Kurths, Norbert Marwan

**Affiliations:** 1 Laboratory of Modelling and Simulation in Engineering, Biomimetics and Prototypes, Faculty of Sciences, University of Yaoundé I, Yaoundé 812, Cameroon; pucicu@gmx.net (A.M.N.); nananbendjo@yahoo.com (B.R.N.N.); 2 Potsdam Institute for Climate Impact Research (PIK), Member of the Leibniz Association, Telegraphenberg, 14473 Potsdam, Germany; eulaliejoelle@yahoo.com (E.K.N.); juergen.kurths@pik-potsdam.de (J.K.); 3 Department of Physics, Humboldt University of Berlin, 12489 Berlin, Germany

**Keywords:** neuron, electric field, weak coupling, gap junction, synchronization, recurrence plot

## Abstract

We investigate the response characteristics of a two-dimensional neuron model exposed to an externally applied extremely low frequency (ELF) sinusoidal electric field and the synchronization of neurons weakly coupled with gap junction. We find, by numerical simulations, that neurons can exhibit different spiking patterns, which are well observed in the structure of the recurrence plot (RP). We further study the synchronization between weakly coupled neurons in chaotic regimes under the influence of a weak ELF electric field. In general, detecting the phases of chaotic spiky signals is not easy by using standard methods. Recurrence analysis provides a reliable tool for defining phases even for noncoherent regimes or spiky signals. Recurrence-based synchronization analysis reveals that, even in the range of weak coupling, phase synchronization of the coupled neurons occurs and, by adding an ELF electric field, this synchronization increases depending on the amplitude of the externally applied ELF electric field. We further suggest a novel measure for RP-based phase synchronization analysis, which better takes into account the probabilities of recurrences.

## 1. Introduction

Action potentials, or spikes, are responsible for the transmission of information through the nervous system [1]. A neuron can generate various temporal patterns of spike signals when it is driven by stimuli or noise from both internal or external environments. Therefore, analyzing spiking patterns of neurons under different stimulations plays an important role in the exploration of the encoding and decoding mechanism of information for neurons. External environmental stimuli in the brain can be of various origins, such as a wide utilization of power lines or electrical equipment. Electromagnetic exposure in the environment today is nearly one hundred times stronger than in previous centuries and many neuronal diseases are probably caused by electromagnetic exposure, as reported by Huang et al. [2]. Experiments with transcranial electrical stimulation have shown that electric field magnitudes in the cortex can be as high as 0.4 mV/mm for a 1 mA stimulation current. For typical electrode configurations used in clinical trials, maximal field intensities of up to 0.8 mV/mm were found when applying 2 mA. More extended areas can reach values of 0.28 mV/mm (95th percentile) under 2 mA stimulation [3,4,5].

An electromagnetic field can affect the neuron sensibility [6,7,8,9]. It also exhibits the excitability of many nerve cells, such as hippocampal cells, or cortical neurons [10,11]. Neurons exposed to an electromagnetic field can change the normal firing properties, which may lead to many neural diseases such as amyotrophic lateral sclerosis, senile dementia, Parkinson’s disease, and Alzheimer’s disease [7,12,13,14].

On the other hand, neurons are strongly coupled in the brain, and they need to synchronize information to encode and decode. Synchronization is a universal concept of nonlinear dynamics [15]. In the brain system, synchronization is a typical form of group motion rhythm, which means the neurons discharge at the same time or their discharge rhythms have at least some kind of relationship [16,17]. Neuronal synchrony activities can be found not only among coupled neuron groups in the same brain region but also among uncoupled neuron groups in the same brain region or among different cortical areas; moreover, synchronization can cross over two hemispheres of the brain [18]. Synchronization processes are crucially important for the neuronal system, and well-coordinated synchrony within and between neuronal populations appears to play an important role in neuronal signaling and information processing.

To study synchronization between neurons, different models of neuron dynamics have been developed, such as the Hodgkin–Huxley (HH) model and all the models derived from it. One of the derived models is the Morris–Lecar (ML) model [19,20]. It has the advantage of exhibiting class I and II neurons. Most studies on neuron synchronization use the Morris–Lecar model under an external electric field. For example, Kitajima and Kurths [21] investigated forced synchronization of electrically coupled class I and class II neurons under different coupling strengths. It was found that class II neurons have a wide parameter region of forced synchronization. However, in general, such studies did not consider the effect of small variations of the coupling strength between neurons.

The assumption of weak neuronal connection is based on the observation that the typical size of a postsynaptic potential is less than 1 mV, which is small in comparison to the mean potential necessary to discharge a cell or the average value of the action potential [22]. In a study of synaptic organization and dynamical properties of weakly connected neuronal oscillators, Hoppensteadt and Izhikevich [23] showed some phase synchronization between neurons in this range of coupling. Moreover, Izhikevich [24] studied the synchronization of elliptic bursters in a range of weak connectivity and found that such weakly connected bursters need few bursts to synchronize and synchronization is possible for bursters having quite different quantitative features. These phenomena were found in different neuron models, such as the FitzHugh–Rinzel, ML, and HH models.

The important question is if, even in the range of small coupling strength, a pair of neurons weakly coupled with gap junction are able to synchronize under the effect of an electric field (EF). Because electromagnetic stimulation can cause many disorders in the neural system, the theoretical investigation of the impact of an external EF on the synchronization of weakly coupled neurons is an important step to understand what happens in the brain during this exposure. Thus, in this work, we study the synchronization of a pair of ML neurons weakly connected with gap junction under an externally applied extremely low frequency (ELF) EF. Here, extremely low frequency means a frequency range between 0 and 10 Hz. Mammalian neurons show intrinsic resonance with frequency selectivity for inputs within the range from 4 to 10 Hz [25,26,27,28,29,30]. Gap junctions (channels that physically connect adjacent cells) provide an efficient and extremely fast way to propagate those signals between neurons [31,32]. In contrast, signal transmissions via chemical synapses have a significant delay (in the order of milliseconds) [33] and are not fast enough to respond to the EF. Therefore, we consider here coupling via gap junction, allowing direct response to the ELF EF. Using recurrence plot-based time series analysis, we investigate how the applied EF affects the condition of synchronization of the coupled neurons. This specific method has the advantage of being able to compare the phases of chaotic weakly coupled systems, even within noncoherent regimes or for spiky signals [34].

Recurrence plots (RPs) represent manifold recurrence features of a dynamical system in phase space [35] and are widely applied in the field of neuroscience [36,37,38,39,40,41,42]. For example, RPs can differentiate the stochastic and deterministic dynamics of irregularly firing cortical neurons [43] and show the average dynamics within a network of synchronized neurons [44] or spontaneous activity in neuronal in vitro cultures [45]. They are also powerful tools to study inter-relationships, coupling directions, phase synchronization, and generalized synchronization [34,46,47,48] and have been applied in different fields, such as chemistry, engineering, physiology, financial markets, and climatology [41,49,50,51,52,53]. Based on EEG measurements, the joint recurrence and the correlation of probability of recurrence were used to reconstruct brain networks [54,55]. A similar approach was used to study the synchronization between neurons based on the Hindmarsh–Rose model [39].

The correlation of probability of recurrence is a commonly used measure for recurrence-based phase synchronization analysis [34,50]. However, this measure is based on Pearson correlation and, thus, has a methodological concern because of the spiky nature of the probability of recurrence.

In this study, we first formulate the mathematical modeling of a single ML neuron and present typical neuron bursting patterns under varying ELF EFs and their corresponding recurrence features as obtained by RPs. We then study the synchronization of two weakly coupled chaotic bursting neurons with and without the influence of an ELF EF. We present the effect of EFs on the mismatch of the mean frequencies of both neurons, even when they are weakly connected. For this purpose, we suggest a slight modification of the recurrence-based phase synchronization measure.

## 2. Model

### 2.1. Morris–Lecar Neuron Model under an Extremely Low Frequency Electric Field

The ML neuron model is a model for electrical activity in the barnacle muscle fiber [19]. It is a simplified version of the HH neuron model for describing the discharge and the refractory properties of real neurons. It can explain the dynamical and biophysical mechanisms of the action potential initiation. This model is chosen as a compromise between a realistic representation of neuronal dynamics and an analytically tractable system. Furthermore, it has an advantage in that the excitability of types I and II can be obtained with a single parameter change. It can also exhibit a variety of bursting types involving regular bursting or irregular bursting and complex bifurcation structures [20,56,57,58].

The ML model has a fast activation variable *v* (membrane voltage) and a slower recovery variable *w*. *v* represents voltage (expressed in mV) and controls the instantaneous activation of fast currents ($i_{\mathrm{fast}}$); *w* is a function of *v* and controls the activation of slower currents ($i_{\mathrm{slow}}$). $c\frac{dv}{dt}$ is the current flowing through the capacitor related to the variation of ionic density between external and internal faces of the membrane. $i_{\mathrm{fast}}$, $i_{\mathrm{slow}}$, and $i_{\mathrm{leak}}$ are ionic currents characterizing the movement of charged particles through the ion channels. This movement of charged particles is due to the opening and closing of each ion channel. $i_{\mathrm{stim}}$ and *c* are the external input current and the membrane capacity, respectively. Finally, this model is given by the following equations:

(1) $$\begin{array}{ccc} c\frac{dv}{dt} & = & i_{\mathrm{stim}}-i_{\mathrm{fast}}-i_{\mathrm{leak}}-i_{\mathrm{slow}} \end{array}$$

(2) $$\begin{array}{ccc} \frac{dw}{dt} & = & \varphi \frac{m_{2}\left(v\right)-w}{b(v)} \end{array}$$

with the currents

$$\begin{array}{ccc} i_{\mathrm{fast}} & = & g_{\mathrm{fast}}m_{1}\left(v\right)\left(v-e_{\mathrm{Na}}\right)\\ i_{\mathrm{slow}} & = & g_{\mathrm{slow}}w\left(v-e_{K}\right)\\ i_{\mathrm{leak}} & = & g_{\mathrm{leak}}\left(v-e_{\mathrm{leak}}\right). \end{array}$$

The parameters $e_{\mathrm{Na}}$, $e_{K}$, and $e_{\mathrm{leak}}$ represent the equilibrium potentials of Na${}^{+}$, K${}^{+}$, and leak ions, respectively, and $g_{\mathrm{fast}}$, $g_{\mathrm{slow}}$, and $g_{\mathrm{leak}}$ are the maximal conductances of the corresponding ion currents. They reflect the ion channels’ densities distributed over the membranes. Control parameter φ is used to control the rate of change of *w*. The steady states $m_{1}$ and $m_{2}$ are nonlinear functions of *v*, given by

(3) $$\begin{array}{ccc} m_{1}\left(v\right) & = & 0.5\left(1+\tanh\frac{v-u_{1}}{u_{2}}\right) \end{array}$$

(4) $$\begin{array}{ccc} m_{2}\left(v\right) & = & 0.5\left(1+\tanh\frac{v-u_{3}}{u_{4}}\right) \end{array}$$

(5) $$\begin{array}{ccc} b(v) & = & \frac{1}{\cosh\frac{v-u_{3}}{2u_{4}}}. \end{array}$$

$u_{1}$ and $u_{3}$ are the activation midpoint potentials at which the corresponding currents are half activated. $u_{2}$ and $u_{4}$ denote the slope factors of the activation. The time constant of the potassium activation is *b*. When a time-varying ELF EF is applied to the brain, it can induce a charge movement in the brain tissue; in which case, the current flow occurs mostly in the extracellular medium [59]. Therefore, an external EF will induce a membrane depolarization Δv which will modulate neuronal bursting behavior. For the sake of simplicity, we consider a steady external sinusoidal electrical field

(6) $$\begin{array}{c} v_{e}=\frac{A}{\omega }\sin\omega t+V_{E} \end{array}$$

where $V_{E}$ is the direct voltage, *A* the amplitude, and ω the frequency of the ELF EF. The field-induced membrane depolarization Δv can be expressed by [60]

(7) $$\begin{array}{c} \Delta v_{}=\frac{A}{\omega }\frac{\sin\omega t-\cos\omega t}{1+{\left(\omega t_{1}\right)}^{2}}+V_{E} \end{array}$$

with $t_{1}$ significantly small and the frequency in the extremely low frequency area $\omega t_{1}\ll 1$. Thereby, Equation (7) can be simplified to

(8) $$\begin{array}{c} \Delta v=\frac{A}{\omega }\sin\omega t+V_{E}. \end{array}$$

According to Equation (8), the sinusoidal EF $v_{e}$ equals its field-induced membrane depolarization Δv. Considering that Δv acts as an additive perturbation to the membrane potential, the dynamics of a neuron during exposure can be described by [61]

(9) $$\begin{array}{ccc} c\frac{dv}{dt} & = & i_{\mathrm{stim}}-\frac{d\Delta v}{dt}-i_{\mathrm{fast}}-i_{\mathrm{leak}}-i_{\mathrm{slow}} \end{array}$$

(10) $$\begin{array}{ccc} \frac{dw}{dt} & = & \varphi \frac{m_{2}\left(v\right)-w}{b(v)} \end{array}$$

with

$$\begin{array}{ccc} i_{\mathrm{fast}} & = & g_{\mathrm{fast}}m_{1}\left(v\right)\left(v+\Delta v-e_{\mathrm{Na}}\right)\\ i_{\mathrm{slow}} & = & g_{\mathrm{slow}}w\left(v+\Delta v-e_{K}\right)\\ i_{\mathrm{leak}} & = & g_{\mathrm{leak}}\left(v+\Delta v-e_{\mathrm{leak}}\right). \end{array}$$

We assume that the synaptic input current $i_{\mathrm{stim}}=0$ in order to study the response of a cortical neuron model exposed to an external sinusoidal field. Throughout this paper, we use the same parameter values for the ML model as explained in Table 1 [62].

### 2.2. Bursting Patterns of a Neuron

**Figure 1:**
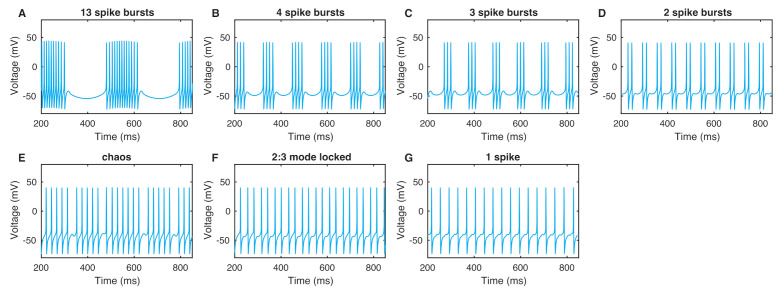
Spiking patterns of ML neuron membrane voltage under an external EF for different frequencies: (**A**) ω=0.02 rad/ms, (**B**) ω=0.05 rad/ms, (**C**) ω=0.06 rad/ms, (**D**) ω=0.10 rad/ms, (**E**) ω=0.286 rad/ms, (**F**) ω=0.32 rad/ms, and (**G**) ω=0.5 rad/ms.

To explore how the neuron model responds to the externally applied ELF EF, we study the dynamics described by Equations (9) and (10) under the sinusoidal stimulus $v_{e}$, Equation (6). The simulations are implemented using the 4th-order Runge–Kutta method with a time step of 0.01 ms. Initial conditions are chosen as the resting values of membrane voltage in the absence of stimuli, that is, v(0)=−65 mV and w(0)=0. The length of the time series is 2000 ms. The response of a neuron induced by an EF depends on the EF’s frequency ω (Figure 1 for ω in the range 0≤ω≤0.5 rad/ms). The amplitude of the external EF is set very small, A=0.1.

**Figure 2:**
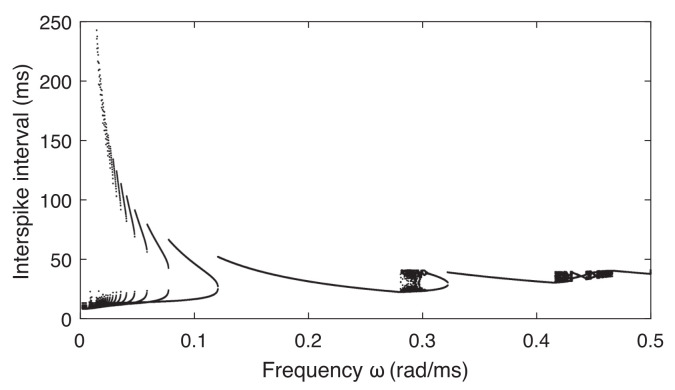
Bifurcation diagram of ML neuron dynamics under an external EF for varying frequencies ω (based on interspike intervals of the membrane voltage). For better visibility of the dynamics for larger ω, the *y*-axis was bounded to 250 ms.

The firing pattern of a neuron stimulated by an external EF varies when changing the frequency ω (Figure 1 and Figure 2). For an ELF EF with very low frequency, the neuron fires periodically. We find *n* spike bursting states, and the number *n* can be large. *n* spike bursting means that we have *n* action potentials in every stimulus period (Figure 1A–D and Figure 2) for ω=[0.001,0.120] rad/ms. After this range ω of *n*-periodic bursting, the neuron bursts synchronously to the stimulus ω=[0.121,0.280] rad/ms. After this range of ω, the neuron exhibits a chaotic response (Figure 1E) with ω=[0.281,0.320] rad/ms where the membrane potential responses are aperiodic and irregular. As ω is further increased, a mode locking pattern of bursting appears (Figure 1F), finally followed by synchronized firing with only one action potential in every stimulus, which can maintain this state for a long-term frequency band (Figure 1G). Neuron dynamics are obviously very sensitive to the frequency of the stimulus by the ELF EF.

## 3. Recurrence Quantification Analysis (RQA)

In the following, neuron dynamics will be studied using recurrence quantification analysis (RQA). This method quantifies certain recurrence features of the dynamical system in its corresponding phase space [35,63]. We define a recurrence of a trajectory $\vec{x}\left(t\right)\in R^{m}$ (with *m* the dimension of the system) of a dynamical system by saying that the trajectory has returned at time t=j to the former point in phase space visited at t=i (with i∈[1,N] and *N* the length of time series) if

(11) $$\begin{array}{c} R_{i,j}=\Theta \left(\varepsilon -∥\vec{x}\left(i\right)-\vec{x}\left(j\right)∥\right) \end{array}$$

where ε is a pre-defined threshold and $\Theta \left(\cdot \right)$ is the Heaviside function. We have a matrix of (0, 1), where 1 at (*i*, *j*) means that $\vec{x}\left(i\right)$ and $\vec{x}\left(j\right)$ are neighbors and 0 means that they are not. The resulting black and white representation of this binary matrix is called a recurrence plot (RP). For the selection of the recurrence threshold ε, different strategies are available, depending on the research question [64,65,66,67,68,69]. Here, we use an approach to select ε in a way that ensures a certain recurrence point density. This allows a better comparability between RPs of different systems [68].

The RP method has been intensively studied and applied in the last years. Different measures of complexity have been proposed that can classify different dynamics, identify dynamical transitions, or detect couplings, causality, or synchronization [35].

If not all state variables of the state vector $\vec{x}$ are available, a phase space reconstruction has to be applied. Here, we use the recently proposed PECUZAL method to reconstruct the phase space trajectories [70]. This method allows us to use multiple embedding delays τ. The embedding parameters are listed in Table 2.

**Figure 3:**
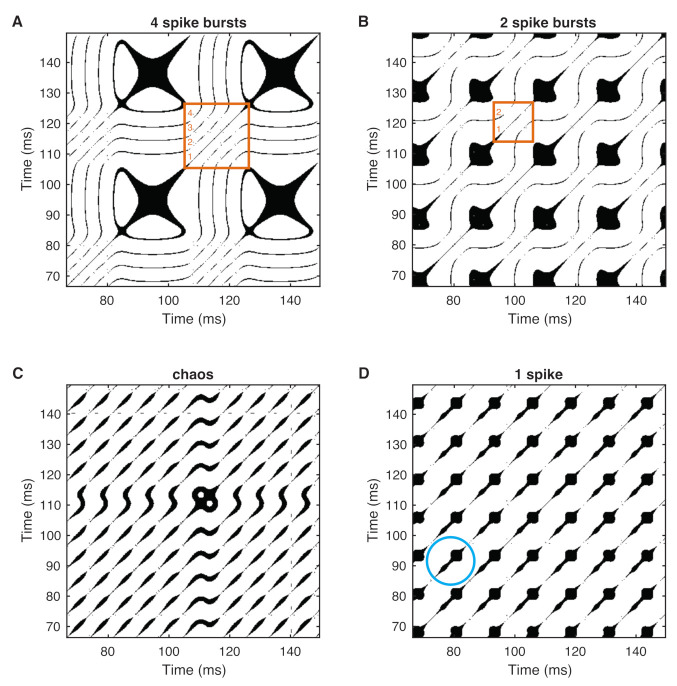
RPs of the membrane voltage *v* of selected bursting neurons: (**A**) 4 spike burst (ω=0.05 rad/ms), (**B**) 2 spike burst (ω=0.10 rad/ms), (**C**) chaos (ω=0.286 rad/ms), and (**D**) 1 spike (ω=0.5 rad/ms). Diagonal lines represent the spikes, the larger extended structures represent the “silent” epochs, and the structures perpendicular to the diagonal lines and small thickenings represent the slow–fast dynamics (blue circle in (**D**)). The orange boxes in (**A**,**B**) mark a sequence of diagonal lines. The number of diagonal lines counted from the main diagonal of such a box towards the corner of this box represents the number of spikes within this period. Recurrence threshold ε is selected to ensure a recurrence point density of 0.15.

RPs of the different bursting neurons represent a typical pattern (Figure 3, using an ε that ensures a recurrence point density of 0.15). Each “dashed-dotted” diagonal line in the RPs corresponds to a spike. For the alternating spiking behavior, we have a set of dashed lines followed by an extended black region (Figure 3A,B). The set of *n* spikes is well distinguished by the number of dashed lines (see some orange boxes marked in the figure). The block-like black region represents the silent state between each stimulus, which is a period for which the neuron cannot respond to a stimulus. On the small scale, the diagonal lines show some additional patterns, i.e., small structures sitting perpendicularly at these lines or thickenings, similar to bumps or knobs. This is a typical feature of slow–fast systems [71].

In order to go beyond the visual impression of the RP, we use recurrence quantification analysis (RQA) [35,72]. The RQA measures are based on the recurrence point density and the diagonal and vertical line structures of the RP. For example, the recurrence point density $\frac{1}{N^{2}}\sum R_{i,j}$ corresponds to the probability that a state will recur. The calculation of this measure can also be restricted to a diagonal-wise calculation, i.e., the recurrence point density along a diagonal with distance τ from the main diagonal $R_{i,i}=1$ [35]. This gives us an estimator of the probability that the system returns to a previous state after time τ and is called the τ-recurrence rate,

(12) $$\begin{array}{c} RR_{\tau }=\frac{1}{N-\tau }\sum_{i=0}^{N-\tau }R_{i,i+\tau } \end{array}$$

where τ is the set time and *N* the total number of points in the phase space. The distance between the peaks in an $RR_{\tau }$ plot corresponds to the period length of oscillations or the interspike intervals of spike trains similar to the neuron’s spiking/bursting patterns.

**Figure 4:**
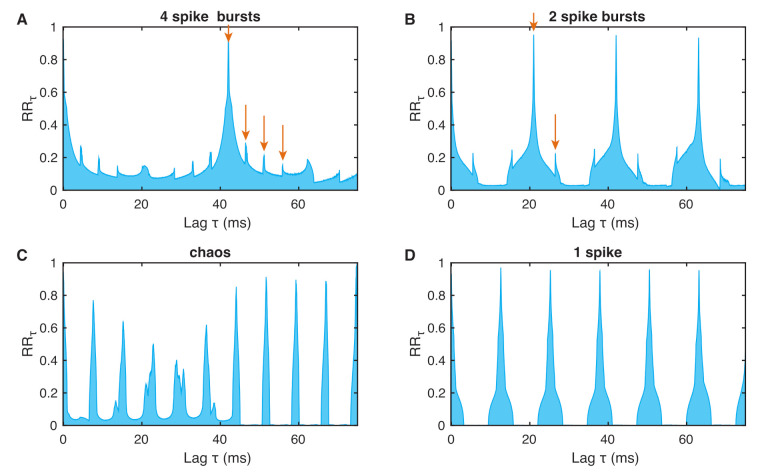
Probability of recurrence after time τ (τ-recurrence rate) for the bursting neurons as shown in Figure 3: (**A**) 4 spike burst, (**B**) 2 spike burst, (**C**) chaos, and (**D**) 1 spike. The *n* bursts are visible as the rather thin side peaks of the main peaks (in addition to the main peak).

The spike trains of 4 spikes, 2 spikes, chaos, and 1 spike have their specific probability distributions for recurrence after lag τ (Figure 4). Where the 1-periodic spike occurrence is clearly visible for 1 spike (Figure 4D), the 2 and 4 spikes produce more subtle probability distributions, revealing different periodicities and large blocks between the bursting periods (Figure 4A,B). The $RR_{\tau }$ of the chaotic bursting exhibits a more complicated distribution of peaks corresponding to the unpredictable occurrence of spikes (Figure 4C).

## 4. Coupling of Two Bursting Neurons

### 4.1. Model and Numerical Simulation

**Figure 5:**
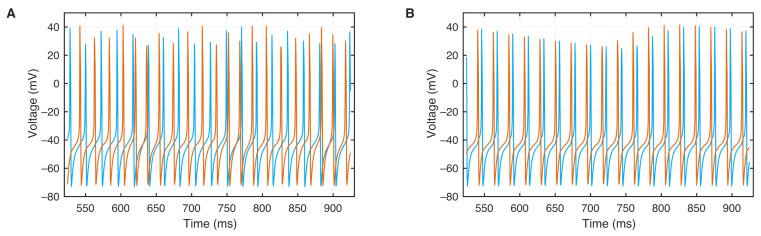
Spiking pattern in the membrane voltage of two weakly coupled neurons in an ELF EF in chaotic regime (ω=0.286) with (**A**) no synchronization with g=0.01 and (**B**) phase synchronization with g=0.04.

A coupling between ML neurons is realized by a gap junction. We suppose that the two neurons are slightly different by considering different values of $u_{2}$ and $u_{3}$ in Equations (4) and (5), i.e., $u_{2,1}=-18.0$ mV and $u_{2,2}=-18.1$ mV, and $u_{3,1}=-12.8$ mV and $u_{3,2}=-10$ mV for neurons 1 and 2, respectively. Moreover, both neurons start using different initial conditions $v_{1}\left(0\right)=-65.6$ mV and $v_{2}\left(0\right)=-60$ mV. We integrate the model for 50,000 time steps (with dt=0.05) and remove the first 10,000 points as transients. Using the ELF EF frequency that leads to chaotic bursting ω=0.286 rad/ms, the coupled chaotic bursting ML neurons under ELF EF exposure can be expressed as

(13) $$\begin{array}{ccc} c\frac{dv_{1}}{dt} & = & i_{\mathrm{stim}}-\frac{d\Delta v_{1}}{dt}-i_{1,\mathrm{fast}}-i_{1,\mathrm{slow}}-i_{1,\mathrm{leak}}-g\left(v_{1}-v_{2}\right) \end{array}$$

(14) $$\begin{array}{ccc} \frac{dw_{1}}{dt} & = & \varphi \frac{m_{2}\left(v_{1}\right)-w_{1}}{b(v_{1})} \end{array}$$

(15) $$\begin{array}{ccc} c\frac{dv_{2}}{dt} & = & i_{\mathrm{stim}}-\frac{d\Delta v_{2}}{dt}-i_{2,\mathrm{fast}}-i_{2,\mathrm{slow}}-i_{2,\mathrm{leak}}-g\left(v_{2}-v_{1}\right) \end{array}$$

(16) $$\begin{array}{ccc} \frac{dw_{2}}{dt} & = & \varphi \frac{m_{2}\left(v_{2}\right)-w_{2}}{b(v_{2})}, \end{array}$$

where *g* is the gap which represents the electrical junction between the neurons. With these two different chaotic neurons, we will now study the phase synchronization between them and focus on the range of weak coupling, i.e., with 500 values of *g* within the interval g=[0,0.15] (Figure 5).

When bursting begins at the same time in the coupled neurons, we have bursting synchronization irrespective of the neurons’ spiking behaviors within a given burst event. From a dynamical point of view, since we assign a phase that increases by 2π at each burst event, we regard bursting synchronization as a kind of phase synchronization [73]. Thus, first we will determine the phase of each chaotic bursting neuron. A frequently used approach to calculate the phase of a signal is using the Hilbert transformation [15]

(17) $$\begin{array}{c} \phi \left(t\right)=\arctan2\left(v_{H}\left(t\right),v\left(t\right)\right) \end{array}$$

where $v_{H}$ is the complex part of the Hilbert transform of the membrane voltage v(t) and ϕ(t) increases continually with time. Since chaotic neurons have chaotic spikes, the phase of chaotic neurons changes also chaotically. Unfortunately, this approach does not work well for spiky signals and can cause slipping of the instantaneous phases. Nevertheless, for long-term averages, it provides useful results.

To detect phase synchronization of chaotic coupled neurons and to evaluate the effect of an ELF EF on this synchronization, we first consider the absolute phase difference between the membrane voltage of both neurons without and with applied EF. Phase synchronization occurs if the difference $\phi_{1}\left(t\right)-\phi_{2}\left(t\right)$ between the phases of the two neurons does not grow with time [74]. This means that the two neurons, on average, generate spikes almost simultaneously. With the knowledge of the phase ϕ(t), the frequency $\bar{\omega }\left(t\right)=\frac{d\phi (t)}{dt}$ and the mean frequency $\Omega =\langle \frac{d\phi (t)}{dt}\rangle$ can be defined. A weaker form of synchronization is frequency locking. Frequency locking between coupled systems can be measured by the mismatch between the average frequencies $\Delta \Omega =\Omega_{1}-\Omega_{2}$, with ΔΩ→0 for phase locking.

**Figure 6:**
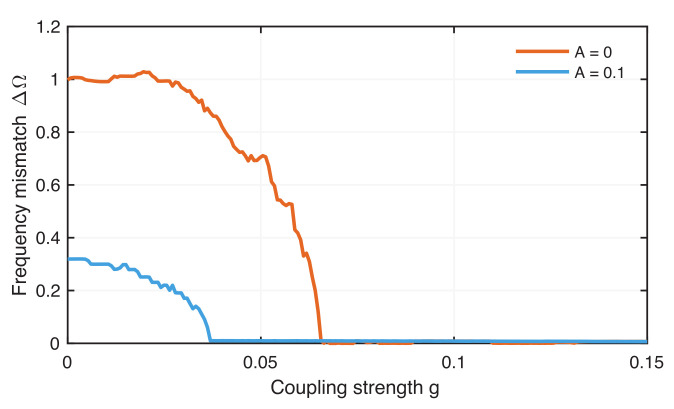
Frequency mismatch between chaotic coupled neurons for increasing coupling *g* without external EF (red) and with external EF where A=0.1 and ω=0.286 (blue).

The weakly coupled neurons show frequency locking without ELF EF when the coupling exceeds a critical value (Figure 6). The frequency mismatch ΔΩ between both neurons is constant between g=0 (no coupling) and g=0.025. After this value, ΔΩ is decreasing and vanishes around g=0.066, indicating the onset of synchronization frequency locking between the neurons.

With ELF EF applied, the frequency difference is smaller, even for g=0, and decreases much faster than without EF; the neurons become frequency-locked for g=0.037 (Figure 6). Thus, in a range of weakly connected neurons, applying an external ELF EF on the chaotic coupled ML neurons enhances frequency-locked synchronization. This confirms earlier findings of synchronized neurons using a different model of weakly connected bursters [24].

**Figure 7:**
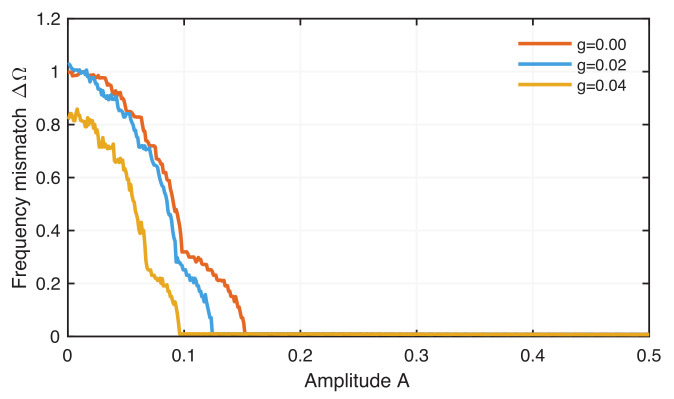
Frequency mismatch between chaotic coupled neurons for increasing amplitude *A* and for different coupling strengths *g*.

Since the firing pattern strongly depends on the amplitude of the ELF EF, we expect that the occurrence of frequency locking also depends on this external stimulus amplitude. In fact, we find that an amplitude value of A=0.15 is strong enough to cause a complete synchronization of two neurons even without coupling (Figure 7). Therefore, we select a lower amplitude value of A=0.1, where we still have a significant frequency mismatch between the uncoupled neurons. A weak coupling between the neurons leads, finally, to frequency-locked synchronization for lower ELF EF amplitudes.

To test for phase synchronization, i.e., whether the difference $\phi_{1}\left(t\right)-\phi_{2}\left(t\right)$ remains constant, we will use an alternative method which can derive the phases of spiky signals in a more reliable way.

### 4.2. Phase Synchronization Analysis Using Recurrence Features

**Figure 8:**
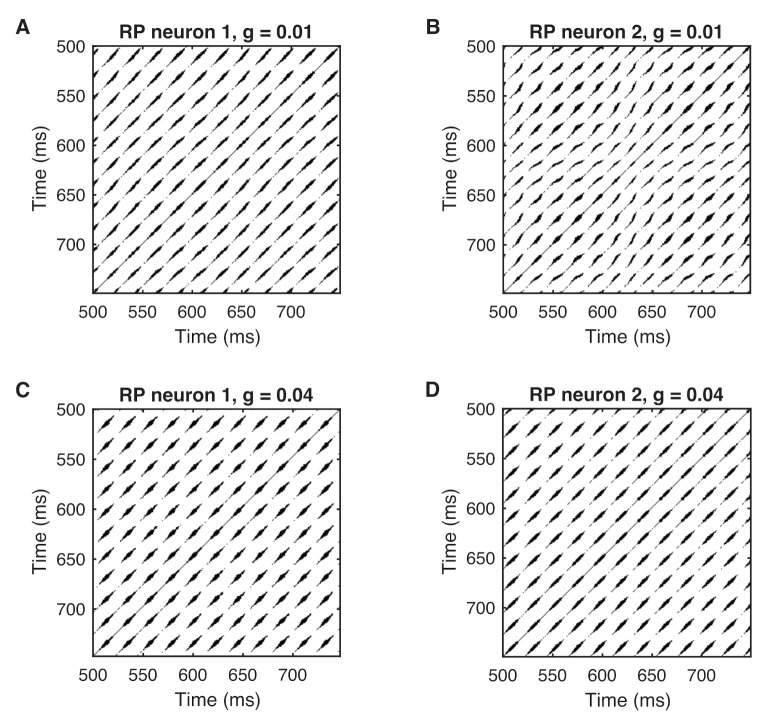
Recurrence plots of the membrane voltage for weakly coupled neurons as shown in Figure 5 for (**A**,**B**) no synchronization, g=0.01, and (**C**,**D**) phase synchronization, g=0.04. Embedding parameters were estimated using the PECUZAL method [70]; the recurrence threshold is selected to ensure a recurrence rate of RR=0.1.

Phase synchronization is related to recurrence of states. Therefore, RPs are a natural tool to study phase synchronization [35]. The spiking pattern causes regular and almost periodic occurrence of diagonal line structures in the RPs (Figure 8). Here, we use a recurrence threshold ε to ensure a recurrence point density of 0.1. Although we notice a certain amount of similarity between the RPs of neuron 1 and neuron 2 in the nonsynchronized regime, we still see deviations in the line patterns of the RP of neuron 2 (Figure 8A,B). In contrast, the RPs of neuron 1 and neuron 2 for the in-phase synchronized regime show a striking similarity (Figure 8C,D).

**Figure 9:**
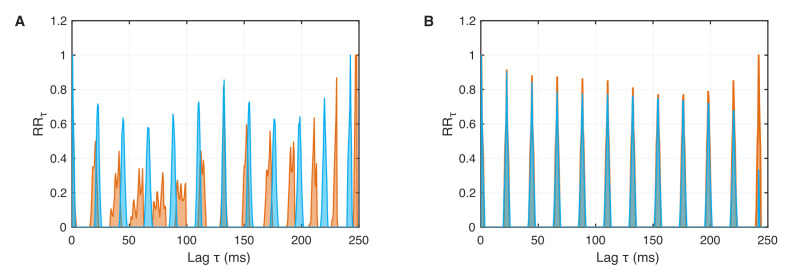
τ-recurrence rate for weakly coupled neurons as shown in Figure 5 and Figure 8 for (**A**) no synchronization, g=0.01, and (**B**) phase synchronization, g=0.04. For phase synchronization, the τ-recurrence rate series for both neurons are almost completely overlapping.

The vertical distance between these diagonal line structures is related to the phase. Therefore, we can use the density of recurrence points along diagonals parallel to the main diagonal, the τ-recurrence rate $RR_{\tau }$, Equation (12), as an estimator of the phase distribution and compare it between different systems. For two nonsynchronized systems, the recurrence probabilities should differ significantly (Figure 9A). During phase synchronization, $RR_{\tau }$ should have high probabilities at the same τ values; thus, the shape of $RR_{\tau }$ should be very similar (Figure 9B). Therefore, $RR_{\tau }$ has been used to construct a measure for phase synchronization between two signals $x_{1}$ and $x_{2}$ by calculating the Pearson correlation of probability of recurrence (CPR) between $RR_{\tau }^{x_{1}}$ and $RR_{\tau }^{x_{2}}$ [34]

(18) $$CPR^{P}=\frac{\mathrm{cov}(RR_{\tau }^{x_{1}},RR_{\tau }^{x_{2}})}{\sigma_{RR_{\tau }^{x_{1}}}\sigma_{RR_{\tau }^{x_{2}}}},$$

with $\sigma_{RR_{\tau }}$ the standard deviation of the corresponding $RR_{\tau }$ series. CPR values of 1 would then correspond to phase synchronization and 0 to no synchronization. Here, it is important to remove the first peak in $RR_{\tau }$ close to τ=0 because these values correspond to the main diagonal in the RP present in all systems [50]. Therefore, this first peak would indicate some kind of similarity between $RR_{\tau }\left(x_{1}\right)$ and $RR_{\tau }\left(x_{2}\right)$ even for completely desynchronized systems. Such exclusion of the first part of the $RR_{\tau }$ series corresponds to applying a Theiler window [75]. Here we used a Theiler window of 25 mS.

Another concern on calculating the CPR measure using Equation (18) is the spiky shape of the $RR_{\tau }$ series, biasing the Pearson correlation estimation. As an alternative, we could use the Spearman rank correlation instead of the Pearson correlation,

(19) $$CPR^{S}=\frac{\mathrm{cov}\left(R\left(RR_{\tau }^{x_{1}}\right),R\left(RR_{\tau }^{x_{2}}\right)\right)}{\sigma_{R(RR_{\tau }^{x_{1}})}\sigma_{R(RR_{\tau }^{x_{2}})}},$$

with the $RR_{\tau }$ series converted to the ranks $R(RR_{\tau })$. This correlation measure is expected to work better for non-normal distributed data, as the $RR_{\tau }$ series would be.

**Figure 10:**
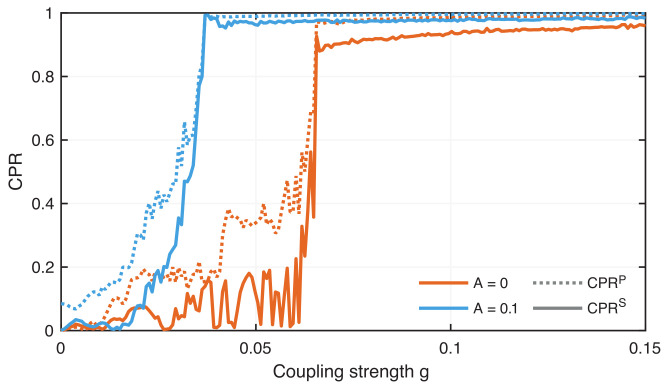
Correlation of probability of recurrence CPR based on Pearson (dotted) and Spearman (line) correlations indicating the onset of phase synchronization between chaotic coupled neurons without external EF (red) and with external EF where A=0.1 and ω=0.286 (blue).

Both CPR measures clearly show the onset of phase synchronization at g=0.066 for neurons without ELF EF and at g=0.037 for neurons with ELF EF (Figure 10). There are some differences between $CPR^{P}$ and $CPR^{S}$. During phase synchronization, $CPR^{P}$ is almost 1, but $CPR^{S}$ is slightly below 1, even more obvious for the coupled neurons without ELF EF. However, for phase synchronization, we would expect to have a CPR value of 1. Moreover, the transition from a nonsynchronized regime to a synchronized regime is not as abrupt as indicated by ΔΩ (Figure 6), but $CPR^{S}$ changes almost abruptly from very low values to very large values, whereby $CPR^{P}$ shows a more gradual increase (and even step-wise increase for A=0). This finding indicates that the Spearman-based CPR is obviously not a better choice than the Pearson-based CPR measure.

The $RR_{\tau }$ series represents probabilities of recurrence. Therefore, it seems more natural to use a measure that can directly quantify the difference between probability populations, such as Kullback–Leibler distance [76] or Hellinger distance [77]. Here we test the use of the Hellinger distance

(20) $$H\left(RR_{\tau }^{x_{1}},RR_{\tau }^{x_{2}}\right)=\frac{1}{\sqrt{2}}∥\sqrt{RR_{\tau }^{x_{1}}}-\sqrt{RR_{\tau }^{x_{2}}}∥,$$

which corresponds to the Euclidean norm of the square root distances between the $RR_{\tau }$ series of the two signals. Values of *H* close to 0 indicate phase transition, whereas values close to 1 indicate nonsynchronized regimes.

To assess whether the variation of *H* indeed reveals phase synchronization, we use a simple block shuffling approach to test the null hypothesis that the signals are not synchronized. Block shuffling splits a time series into a number of blocks (here, we used five blocks) of equal width at random indices and randomly concatenates these blocks to create a new surrogate time series. Such surrogates preserve short-term temporal properties but destroy long-term dynamical information and, thus, correlations when compared with another signal. The distribution p(H) derived from the ensemble of surrogates is then used to define the confidence limit of 95% (simply by using the 95% quantile of this test distribution p(H)). Considering A=0, we find the confidence limit by $H_{0.95}$ as 0.17. Values of *H* below this value can be considered to represent phase synchronization.

**Figure 11:**
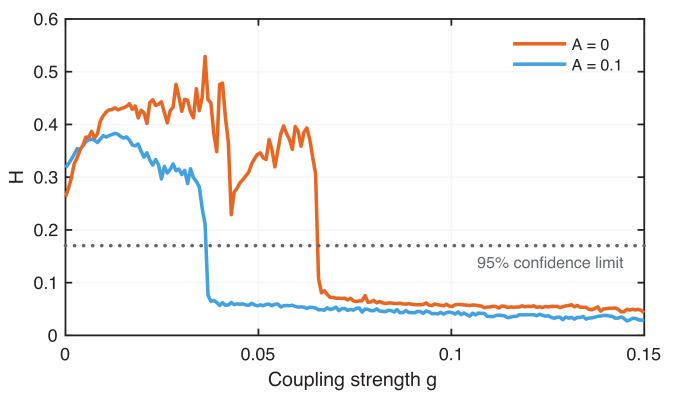
Hellinger distance of the τ-recurrence rate indicating the onset of phase synchronization between chaotic coupled neurons without external EF (red) and with external EF where A=0.1 and ω=0.286 (blue). A drop of *H* below the confidence limit of 95% (dotted line) represents the significance of this finding.

The measure *H* indicates the transition from the nonsynchronous to the phase synchronization regime of the two weakly coupled neurons (Figure 11). The change in *H* is significant. Moreover, the variation in *H* over increasing coupling strength *g* reveals the more gradual change to the phase synchronization as well as the step-like transition to phase synchronization for the situation without ELF EF caused by phase jumps.

## 5. Conclusions

The synchronization of weakly coupled Morris–Lecar neurons under common external forcing has been studied previously. For example, Kitajima and Kurths [21] used interspike intervals (to study frequency locking) and Yi et al. [62] considered the average firing rate. In general, the numerical calculation of the phases of spiky signals using the Hilbert transform is problematic. An alternative way to identify the phases in dynamical systems is to use recurrence plots [34]. This method can find phases for noncoherent and spiky signals. We, therefore, used a recurrence-based approach, which decodes the phase in terms of specific recurrence patterns in the recurrence plot, and demonstrated its potential for the study of spiking patterns of neurons.

In this work, the spiking patterns of Morris–Lecar neurons under ELF sinusoidal EF and the synchronization of two neurons weakly coupled with gap junction under ELF EF were investigated using this recurrence-based approach. The representation of the dynamics of the neurons’ membrane voltages by recurrence plots provided a convenient approach to compare the recurrence features of their spiking patterns. Various spiking patterns, such as periodic and chaotic bursting and periodic spikes, were observed. The spiking patterns were found to be very sensitive to changes of the stimulus frequency.

Moreover, the recurrence approach allows us to consider phase differences between the spiking patterns in a more robust way than the frequently used Hilbert transform. We have introduced an alternative measure for testing phase synchronization using recurrences. Instead of comparing the probabilities of recurrences (as represented by the τ-recurrence rate) by correlation coefficients, we suggest to use the Hellinger distance as a more natural measure because it quantifies the differences between probabilities. The typically used Pearson correlation is biased because the τ-recurrence rate does not follow a normal distribution. The Spearman rank correlation could be an alternative, but we found additional bias due the large number of zeros in the τ-recurrence rate series.

By using recurrence-based synchronization measures, we found that even without external EF, phase synchronization of two ML neurons can occur for a range of values of coupling strength. Moreover, phase synchronization can be enhanced by an additional external EF. This physiological behavior might be of importance for the functioning of the brain when exposed to electromagnetic fields, such as by power lines, electrical equipment, or cellular radio towers.

## Figures and Tables

**Table 1:** Parameters used for the ML model.

$u_{1}$	−1.2 mV	$g_{\mathrm{fast}}$	20 mS/cm${}^{2}$	$e_{\mathrm{Na}}$	50 mV	φ	0.15
$u_{2}$	18 mV	$g_{\mathrm{slow}}$	20 mS/cm${}^{2}$	$e_{K}$	−100 mV	*c*	2 μ
$u_{3}$	−13 mV	$g_{\mathrm{leak}}$	2 mS/cm${}^{2}$	$e_{\mathrm{leak}}$	−70 mV	$V_{E}$	−17.63 mV
$u_{4}$	10 mV						

**Table 2:** Embedding parameters indicated by the PECUZAL algorithm.

Time Series	Dimension	Delay
4 spike burst (ω=0.05 rad/ms)	3	17, 22
2 spike burst (ω=0.10 rad/ms)	2	16, 20
chaos (ω=0.286 rad/ms)	2	20
1 spike (ω=0.5 rad/ms	2	19

## Data Availability

Code used to perform the analysis of this study is available via Zenodo, doi:10.5281/zenodo.5910812 (accessed on 29 December 2021).

## Abbreviations

The following abbreviations are used in this manuscript:

CPR	correlation of probability of recurrence
EF	electric field
ELF	extremely low frequency
HH	Hodgkin–Huxley model
ML	Morris–Lecar model
RP	recurrence plot
RQA	recurrence quantification analysis
RR	recurrence rate
